# Lung Ultrasound: A Leading Diagnostic Tool

**DOI:** 10.3390/diagnostics13101710

**Published:** 2023-05-12

**Authors:** Marcello Demi, Gino Soldati

**Affiliations:** 1 Department of Bioengineering, Fondazione Toscana Gabriele Monasterio, 56126 Pisa, Italy; 2 Diagnostic and Interventional Ultrasound Unit, Ippocrate Medical Center, 55032 Lucca, Italy; soldatigino@yahoo.it

Thoracic ultrasound is an important diagnostic tool employed by many clinicians in well-defined applications. Over the last ten years, many technical, methodological, and clinical questions have been clarified, even though some problems await a response from the scientific medical and non-medical community. Its use is prominently clinical; that is, thoracic ultrasound is a diagnostic tool that combines classical semiotics and diagnostic reasoning. Equally evident is the content of the information provided by this tool, constituting a mix of classical anatomical information and information on the presence, distribution, and shape of acoustic traps that alter the physical state of the lung’s surface. There is no doubt that the ultrasound manifestations of so-called interstitial syndrome represent a new field yet to be fully explored given the relationship between the superficial density of the lung when still aerated and under pleural histopathology.

Six research papers and two review papers on the clinical applications of lung ultrasound are presented in this Special Issue. In [1], 101 unselected pulmonary patients were evaluated blindly with ultrasound chest examinations. The obtained results show how chest ultrasound is an effective complementary diagnostic tool for pulmonologists. However, some discordances regarding the usefulness of LUS must also be considered. For example, the value of B-Line scores (BLSs) in guiding fluid management during critical illness [2] showed that daily BLS monitoring did not lead to a different cumulative fluid balance in surgical ICU patients compared to standard care. The B-line score, i.e., the simple counting of B lines, may not be a significant parameter. In [3], the authors show how a simple pulmonary assessment using LUS provides relevant information about pulmonary congestion in hemodialysis patients (outperforming chest radiography) and identifies patients at risk of complications. The authors suggest that dialysis units adopt LUS in their daily clinical practice as a bedside tool not only for fluid status assessment and dry weight prescription but also to prevent intradialytic hypotension and drive ultrafiltration prescription during the whole hemodialytic session. The authors of [4] show how US offers good sensitivity in the detection of pleural abnormalities localized in the costo-phrenic angle (CPA) and how an accurate ultrasound examination of CPA in patients affected by pleural effusion or suspected malignant pleural effusion could assess even millimetric pathological lesions not easily detectable by chest CT scan. In [5], the role of ultrasound in the diagnosis of pulmonary infection, caused by intracellular fungal pathogens or mycobacteria, is analyzed through a systematic review. Lung ultrasound can also be successfully used to examine children [6,7] and newborns [8]. The authors of [6] describe protocols for LUS examinations of children, discuss diagnostic criteria, and introduce methods for the diagnosis and classification of pulmonary diseases commonly encountered in pediatric cardiology. According to the authors’ judgement, US is an easy, accurate, rapid, inexpensive, and radiation-free tool for the diagnosis and follow-up of major pulmonary complications in pediatric cardiac surgery, and they strongly encourage its use in routine practice. Cystic fibrosis (CF) lung disease is analyzed in [7]. The aim of the study was to evaluate a newly conceived LUS score by comparing it to the modified Bhalla CT score. The results show that LUS score can be used in the diagnosis and monitoring of CF lung disease in children. In [8], the use of lung ultrasonography and the LUS score is suggested not only for the initial diagnosis but also for the monitoring of newborns with respiratory problems. Lung ultrasonography appears to be a valuable tool for the real-time assessment of improvement in lung status after starting respiratory support. Therefore, it is an aid for the clinician to adjust management and subsequent support accordingly, increasing or decreasing the latter as needed.

Given the temporal context in which this Special Issue has been proposed, contributions regarding COVID-19 were expected. Three review papers and three research papers were submitted and accepted after revision. COVID-19-associated pneumonia can give rise to a variety of pathological pulmonary changes ranging from mild diffuse alveolar damage (DAD) to severe acute respiratory distress syndrome (ARDS), with the latter being characterized by peripheral bilateral patchy lung involvement. These findings have been well described in CT imaging and anatomopathological cases. Consequently, ultrasound artifacts and consolidations are expected signs in COVID-19 pneumonia because edema, DAD, lung hemorrhage, interstitial thickening, hyaline membranes, and infiltrative lung diseases, when they arise in a subpleural position, generate ultrasound findings. In [9], the structure of the ultrasound images in the normal and pathological lung is analyzed. Lung ultrasound is suggested to play an important role in this context due to its high diagnostic sensitivity, low cost, and simplicity of execution. Despite computed tomography being the gold standard of imaging, lung ultrasound is essential in every situation where CT is neither readily available nor applicable. In [10], the role of lung ultrasound (LUS) in the diagnosis and prognosis of SARS-CoV-2 pneumonia is discussed through a comparison with High-Resolution Computed Tomography (HRCT). In [11], the considerable versatility of LUS in diagnosis, the framing of the therapeutic route, and follow-up for SARS-CoV-2 interstitial syndrome is highlighted. In [12], the presence of LUS artifacts after SARS-CoV-2 infection in children were evaluated, and the associations between the time elapsed since infection and symptomatology during acute infection were analyzed. In a pilot study [13] of post-COVID syndrome patients, the usage of lung ultrasound in a follow-up was evaluated by identifying the variation of reverberation artifacts over the course of approximately one year. In [14], the utility of lung ultrasound with respect to neonates diagnosed with COVID-19 is assessed. The authors suggest that lung ultrasound is a useful diagnostic tool that offers a non-invasive, easy-to-use, and reliable method for lung lesion detection in neonates.

The challenges and potentialities correlated with the use of LUS as a leading diagnostic tool are also addressed by one research paper and two review papers. In [15], hospitalist perceptions of barriers to lung ultrasound adoption in diverse hospital environments are analyzed. The hospitalists interviewed perceive LUS as a tool offering important benefits for patients, clinicians, and health systems. However, the time required to master and perform LUS was perceived to be an important barrier to its adoption. In [16], the authors reveal how hand-held ultrasound devices, which are accessible and comparatively easy to decontaminate, could constitute a reliable tool for evaluating peripheral lung diseases. The study highlights how this tool can be successfully employed as an alternative to repeated X-ray examinations for peripheral lung disease monitoring. In [17], the authors show how current communication technologies can be exploited to allow patients to perform US assessments of their lung status.

Moreover, four research papers and two review papers focused their attention on the analysis and understanding of the acoustic information provided by LUS. A preliminary attempt to overcome the oversimplified B-Lines score uniquely based on the number of observed B-lines is illustrated in [18]. This study concerns the application of lung ultrasound for the evaluation of the significance of both vertical artifact changes with frequency and pleural line abnormalities in differentiating pulmonary edema from pulmonary fibrosis. The mechanisms underlying vertical artifacts in lung ultrasound and their potential use for differentiating cardiogenic pulmonary edema from lung diseases are discussed in [19] and in [20]. In [19], the authors recount the theory of the acoustic trap and the basic research studies supporting the theory. In their contribution, the authors underline how published studies and pilot experiments indicate that the clarification of the relationship between the length and intensity of vertical artifacts and the physical or acoustic composition of sources may be useful for differentiating cardiogenic pulmonary edema from lung diseases. In [20], the author stresses a similar problem. This study highlights an important point: in order to derive further information from the visual inspection of vertical artifacts, the mechanisms that control artifact formation must be identified. In this paper, the link between the visual characteristics of the vertical artifacts (the observed effect) and the distribution of the aerated spaces at the pleural level (the cause) is addressed. Vertical artifacts are frequently seen in a variety of lung diseases, and they vary in length, width, shape, and internal reverberations. The reason for this diversity is still partially unknown and has generated debates between clinicians and physicists. In [21], the most common clinician observations are summarized and explained. The paper underscores the importance of the visual analysis of vertical artifacts along with the importance of strong collaboration between clinicians and physicists. In [22], a finite-element numerical model is proposed to simulate the radio frequency (RF) signals received by a probe when an US pulse is reflected by an acoustic trap that affects a normal lung surface. The RF signals give rise to images of horizontal A-lines and vertical B-lines that are reasonably similar to those observed in real images. The proposed model is useful for studying the impact of a specific lung infiltration on the appearance of LUS images. The authors of [23] describe a systematic working method that was used to comprehend the genesis of the vertical artifacts and their relationship with the surface histopathology of the lung. In this study, the acoustic trap theory is summarized, and the acoustic traps are seen as secondary sources of ultrasound. Moreover, the authors relate that they disagree with the term artifact since it does not adequately represent the informational content of acoustic signs, which, in their opinion, are not artifacts but pathological footprints and anatomical information.

When this Special Issue was proposed, the expected content was stratified into eight topics: (1) the essential physics of lung ultrasound imaging; (2) diagnostic signs provided by lung ultrasound; (3) guidelines in clinical care practice; (4) the biological effects of pulsed ultrasound; (5) theoretical and physical lung modeling; (6) visual and computer-aided image analysis; (7) spectral analysis of radiofrequency (RF) signals; and (8) clinical and open ultrasound platforms. Aside from biological effects and computer-aided image analysis, the received contributions address almost all these topics to varying degrees. On the one hand, many studies warn of the potential biological damage induced by the ultrasound-based examination of the lungs. However, on the other hand, physicians perform lung ultrasound examinations daily, and—to the best of our knowledge—lung damage has never been reported. In most papers reporting damage during lung ultrasound exams, hemorrhages were locally provoked on the lungs of small animals via static long expositions and often by locating the probe directly on the parietal pleura, i.e., by adopting working conditions that are never met in standard lung ultrasound exams. The submitted contributions have demonstrated the solid confidence of physicians to be using a safe [1,3,8,10,13,14] and harmless [8,10,13] imaging device that is free of adverse effects [5] and produces no side effects [12,17]. Computer-aided image analysis is a wide topic ranging from simple algorithms for the detection and counting of vertical artefacts in single-lung US images to convolutional neural networks for an explicit diagnosis of a pathology. Even though many papers in the literature follow this direction, in our opinion, the diagnostic utility of these tools is modest. The manuscripts published in this Special Issue strongly highlight the necessity of understanding the genesis of artefactual information as a unique method of completely and efficiently exploiting this information.

## Data Availability

Not applicable.
